# Chronic relapsing inflammatory optic neuropathy (CRION): a manifestation of myelin oligodendrocyte glycoprotein antibodies

**DOI:** 10.1186/s12974-018-1335-x

**Published:** 2018-10-31

**Authors:** Haeng-Jin Lee, Boram Kim, Patrick Waters, Mark Woodhall, Sarosh Irani, Sohyun Ahn, Seong-Joon Kim, Sung-Min Kim

**Affiliations:** 1 Department of Ophthalmology, College of Medicine, Seoul National University, 101 Daehak-Ro, Jongno-Gu, Seoul, 110-744 Republic of Korea; 2 Department of Neurology, College of Medicine, Seoul National University, 101 Daehak-Ro, Jongno-Gu, Seoul, 110-744 Republic of Korea; 3 Nuffield Department of Clinical Neurosciences, John Radcliffe Hospital, Oxford, UK

**Keywords:** Myelin oligodendrocyte glycoprotein antibodies, Chronic relapsing inflammatory optic neuropathy, Optic neuritis, Multiple sclerosis, Neuromyelitis optica

## Abstract

**Background:**

Key clinical features of chronic relapsing inflammatory optic neuropathy (CRION) include relapsing inflammatory optic neuritis (ON) and steroid dependency, both of which have been reported among patients with myelin oligodendrocyte glycoprotein antibodies (MOG-Abs). We investigated the relevance of the presence of serum MOG-IgG with the current diagnostic criteria for CRION among patients with idiopathic inflammatory optic neuritis (iON).

**Methods:**

Retrospective reviews of a database prospectively collated between 2011 and 2017 from the tertiary referral center for multiple sclerosis and neuromyelitis optica were performed. Sixty-four patients with iON, who did not meet the diagnostic criteria for multiple sclerosis, neuromyelitis optica (NMO) spectrum disorder with/without NMO-IgG, or acute disseminated encephalomyelitis and who had no symptomatic central nervous system (CNS) lesions other than on the optic nerve, were included from a cohort of 615 patients with inflammatory demyelinating diseases of the CNS. Fulfillment of the current diagnostic criteria for CRION, assay results for the serum IgG1 MOG-Ab, and characteristics of CRION patients with MOG-IgG were compared to those of non-CRION patients with MOG-IgG.

**Results:**

Twelve iON patients fulfilled the current diagnostic criteria for CRION, 11 patients were positive for MOG-IgG, and one patient was borderline. Among the other 52 iON patients not meeting the criteria for CRION, 14 had relapsing disease courses and 38 had monophasic courses, of which MOG-IgG positivity were 0% and 29%, respectively. CRION patients with MOG-IgG had more relapsing disease courses (first steroid-dependent worsening/relapse in 2.3 months, range 0.4–7.0) and poorer optical coherence tomography outcomes at follow-up than non-CRION patients with MOG-IgG. However, patients in the two groups did not differ in terms of age of onset, sex, or steroid treatment duration after initial attack.

**Conclusions:**

CRION, according to the current diagnostic criteria, is a relapsing optic neuritis associated with MOG-IgG. Among iON patients with MOG-IgG, the absence of steroid-dependent attacks in the early stages of the disease may predict a long-term non-relapsing disease course and a more favorable outcome.

**Electronic supplementary material:**

The online version of this article (10.1186/s12974-018-1335-x) contains supplementary material, which is available to authorized users.

## Background

Chronic relapsing inflammatory optic neuropathy (CRION), initially described in 2003 [1], is a form of recurrent optic neuritis (ON) that has relatively good response/dependency to steroid treatment. Petzold et al. [2] reviewed 122 case reports and proposed the diagnostic criteria of CRION by adding radiological and laboratory findings: enhancement of the optic nerve on magnetic resonance imaging (MRI) and the absence of aquaporin-4 antibodies (AQP4-Ab).

The antibody to myelin oligodendrocyte glycoprotein (MOG-Ab) has been proposed as a new marker of inflammatory demyelinating diseases (IDDs) of the central nervous system (CNS) [3, 4]. Characteristics of MOG-IgG-associated diseases include optic neuritis as a major symptom, good response to steroid, absence of serum AQP4-Ab, and steroid-dependent relapse or disease exacerbation [4, 5]. Although the features of MOG-IgG-associated diseases are similar to those of CRION, the relevance of the current CRION diagnostic criteria with MOG-IgG seropositivity has not been well studied.

In the present study, we investigated the frequency of CRION and MOG IgG1 antibodies in patients with isolated ON (iON) and compared CRION and non-CRION-MOG-IgG-positive patients.

## Methods

### Subjects

**Fig. 1:**
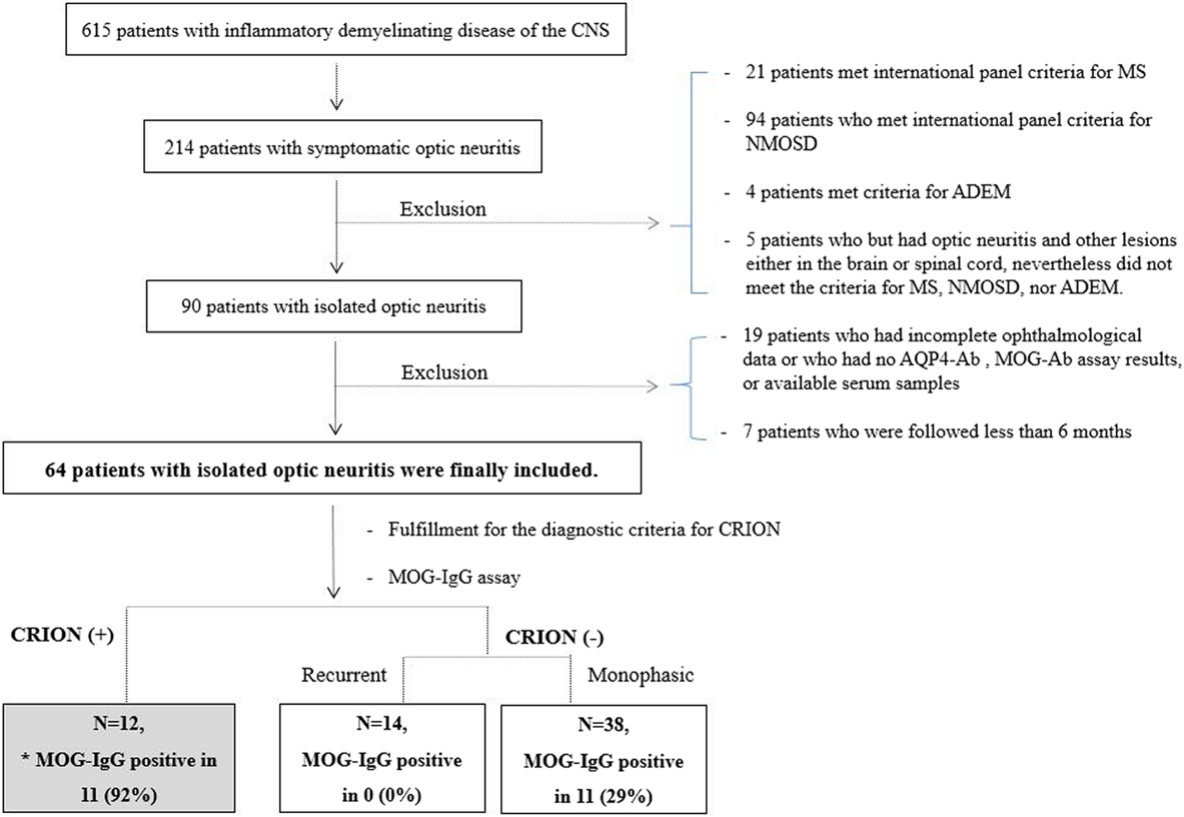
Flow chart of patients with optic neuritis. Patients were classified according to the results of clinical diagnosis and serological status of myelin oligodendrocyte glycoprotein antibodies. *The MOG-IgG result of one chronic relapsing inflammatory optic neuropathy patient, who did not test positive for MOG-IgG, was borderline

A total of 615 consecutive patients identified between 2011 and 2017 with IDDs of the CNS were screened from the prospectively collated database of Seoul National University’s Multiple Sclerosis/Neuromyelitis Optica Center in South Korea. The diagnosis, clinical characteristics, and laboratory findings of patients were reviewed by HJL and SMK. Patients with acute visual symptoms were evaluated by an experienced neurologist and an ophthalmologist. A diagnosis of ON was made on the basis of acute visual symptoms such as decreased visual acuity or visual field defect and evidence of an afferent pupillary defect in the affected eye. MRI imaging and blood sampling were performed. Of the 214 patients with symptomatic ON, patients with multiple sclerosis [6], neuromyelitis optica spectrum disorder (NMOSD) with or without AQP4-IgG [7], acute disseminated encephalomyelitis [8], and patients with symptomatic CNS lesions other than on the optic nerve were excluded from this study. The remaining patients, who were not excluded, were diagnosed as isolated ON. Ninety patients with iON were identified. Subsequently, patients who had incomplete ophthalmological data, e.g., missing optic disc evaluation, measurement of visual acuity, or visual field examination, patients with no AQP4-IgG or MOG-IgG1 assay results or available serum samples, and patients who were followed for less than 6 months were also excluded (Fig. 1). Assays for AQP4-IgG were performed by an in-house fluorescence-activated cell sorting (FACS) assays using live cells expressing human M23 AQP4, as described previously [9, 10]. Finally, 64 patients with iON, seronegative for AQP4-IgG, were included in this study.

### Diagnostic criteria of CRION

Patients were diagnosed with CRION according to recent criteria [2]. It requires at least one relapse, objective evidence for the loss of visual function, seronegative AQP4-IgG results, contrast enhancement of the optic nerve on MRI, response to steroid treatment, and steroid-dependent relapse [2].

### MOG-IgG assays

Of the 64 sera from patients with iON, 33 sera were tested for serum IgG1 MOG-IgG by MW at John Radcliffe Hospital (Oxford, UK), using cell-based assays expressing full-length human MOG [11]; and 38 sera were tested at Seoul National University (Seoul, Korea) by BRK, using flow cytometry (FACS). Seven serum samples were tested at both locations. Epidemiological, clinical, and radiological data were blinded to both MW and BRK until the assays were completed. The flow cytometric MOG-IgG assays were performed with a minor modification from the previously published method [11]. Briefly, human embryonic kidney 293 T cells were transfected with a pcDNA3.1 vector (Invitrogen) containing full-length human MOG (α1, NM_206809.3) cDNA, using lipofectamine. Patient sera were tested at 1:50 dilution, and the mouse anti-human IgG1 Hinge-FITC (hinge) from SouthernBiotech (9052-02, Birmingham, AL) was used at 1:100 dilution. The geometric mean fluorescence (G-mean) ratio was calculated for each patient using the formula: G-mean values of the patient/G-mean values of the healthy control (Additional file 1). The cutoff was set to six standard deviations (SD) [12] above the average. The average G-mean ratio was calculated from 89 controls (39 patients with AQP4-IgG-positive NMOSD, 23 with multiple sclerosis, seven with malignancy, four with polyneuropathy, four with vascular disease, two with dermatomyositis, two with Churg-Strauss syndrome, and one each with CNS lupus, compressive myelopathy, fungal sinusitis, Leber’s hereditary optic neuropathy, neuro-Behcet’s disease, somatoform disorder, hereditary spastic paraplegia, and syringomyelia). As the average G-mean ratio of our 89 control sera was 0.98 ± 0.11 (0.25–1.23) {mean ± SD (minimum–maximum)}, a G-mean ratio above 1.64 was considered positive for MOG-IgG1-Abs. The G-mean ratio values between the highest values of the controls and cutoff values (1.23–1.64) were considered to be a borderline result. Among our 64 patients with iON, the percent positivity of the MOG-IgG assay was similar between Oxford and Seoul, at 39% (13/33) and 32% (12/38), respectively (seven sera were tested both in Oxford and Seoul with identical results).

### Ophthalmological findings

A Snellen chart was used to measure the visual acuity (VA) and converted into logMAR for analysis. Severe visual disability was defined as VA less than 20/200 at nadir. A favorable visual outcome was defined as VA more than 20/40 at final follow-up. Visual fields were examined within 1 month of the first ON attack using the Goldmann or Humphrey perimetry.

Retinal nerve fiber layer, ganglion cell-inner plexiform layer, and macula thicknesses were measured using spectral-domain optical coherence tomography (Cirrus HD OCT, Carl Zeiss Meditec, Inc., Dublin, CA, USA) at least 1 month after intravenous steroid pulse therapy.

### Radiological findings

MRI studies included axial, coronal, and sagittal images of the brain and spinal cord obtained using T1-weighted (W), T2-W, and T1-W fat suppression post-contrast sequences within 1 month of the acute attack. The presence of optic nerve enhancement on MRI was evaluated and analyzed by dividing the anatomical segments of the optic nerve into orbital, canalicular, intracranial, chiasmal, and optic tract regions [13]. On axial images, the proportion of optic nerve enhancement in the orbital area was calculated as follows: the length of enhancement at the maximum length of the orbital optic nerve divided by the maximum total length of the optic nerve in the orbital area. Perineural enhancement [4], defined as extensive enhancement to the soft tissues surrounding the intraorbital portion of the optic nerve, was also evaluated.

### Treatment and disease course

All patients received intravenous methylprednisolone (250 mg every 6 h for either 3 or 5 days). At the discretion of clinicians, oral prednisone was administered and tapered after IV steroid pulse treatment. The average duration of oral steroid use was 34.4 ± 31.8 days. A good response to steroid therapy was defined as the recovery of VA to more than 20/40, within 1–2 months, after steroid pulse therapy. Steroid dependency, based on previously reported characteristics of CRION [1], was defined as a relapse or exacerbation of ON, occurring within 2 months from the time of cessation or dose reduction of steroid treatment. Relapse was defined as a new ON episode lasting at least 24 h. During the follow-up period, the total number of ON attacks and the time interval between attacks were evaluated.

### Standard protocol approvals

The study was approved by the Institutional Review Board of Seoul National University Hospital in Korea (approval number H-1012-080-344). The study protocol followed the tenets of the Declaration of Helsinki.

### Statistical analysis

As the data showed abnormal distribution, non-parametric methods including the Mann-Whitney test and Fisher exact test were used. For all tests, *p* < 0.05 was considered significant. Statistical analyses were performed using SPSS software (version 23 for Windows; SPSS, Chicago, IL, USA).

## Results

### General cohort features

Sixty-four patients with iON were included in the present study (Fig. 1). The mean age of the first ON attack was 42.5 ± 16.6 years. Thirty-six patients (56%) were females. The mean time interval between onset of ON and steroid treatment was 6.5 ± 2.5 days. The total average number of ON attacks was 1.8 ± 1.3 during the total follow-up duration of 39.5 ± 42.3 months (range 6–250). Twenty-six patients had relapsing disease courses, and 38 had monophasic courses.

### Fulfillment of the criteria for CRION and seropositivity to MOG-IgG assay

**Fig. 2:**
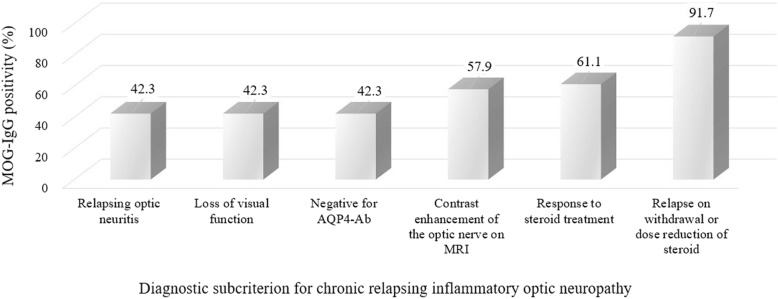
MOG-IgG positivity of each subcriterion for chronic relapsing inflammatory optic neuropathy in relapsing optic neuritis. The positive predictive value of steroid dependency for MOG-IgG was 91.7%, which is the highest predictive factor of MOG-IgG positivity

Of the 26 patients with recurrent iON, 12 patients fulfilled the current diagnostic criteria for CRION; 11 patients were positive for MOG-IgG, and 1 patient was borderline (average G-mean ratio 4.1, range 1.59–7.87) (Additional file 2). Fourteen patients with relapsing iON did not meet the criteria for CRION, due to the absence of either relapse on withdrawal or dose reduction of steroid treatment (*n* = 14), lack of response to steroid treatment (*n* = 6), or absence of contrast enhancement of the optic nerve on MRI (*n* = 7) (Table 1). Among the subcriteria for CRION, the presence of the steroid dependency (relapse on withdrawal or dose reduction of steroid) had the highest positive predictive values (91.7%) for MOG-IgG positivity (Fig. 2).

**Table 1:** Clinical features of patients with recurrent isolated optic neuritis divided according to the subcriteria of chronic relapsing inflammatory optic neuropathy

Patient no.	Sex/age at onset	Criteria for CRION						MOG-IgG assay results	Brain lesion on MRI
		Relapsing optic neuritis	Loss of visual function	Negative for AQP4-Ab assay	Contrast enhancement of the optic nerve on MRI	Response to steroid treatment	Relapse on withdrawal or dose reduction of steroid treatment		
1	M/46	Yes	Yes	Yes	Yes	Yes	Yes	Positive	No
2	F/62	Yes	Yes	Yes	Yes	Yes	Yes	Positive	No
3	F/52	Yes	Yes	Yes	Yes	Yes	Yes	Positive	No
4	M/52	Yes	Yes	Yes	Yes	Yes	Yes	Positive	Nonspecific white matter abnormality
5	M/38	Yes	Yes	Yes	Yes	Yes	Yes	Positive	No
6	F/32	Yes	Yes	Yes	Yes	Yes	Yes	Positive	No
7	F/20	Yes	Yes	Yes	Yes	Yes	Yes	Positive	Brain stem lesion
8	F/23	Yes	Yes	Yes	Yes	Yes	Yes	Positive	No
9	F/51	Yes	Yes	Yes	Yes	Yes	Yes	Positive	No
10	F/39	Yes	Yes	Yes	Yes	Yes	Yes	Positive	No
11	M/41	Yes	Yes	Yes	Yes	Yes	Yes	Positive	Asymptomatic focal T2 high signal intensity in the pontine tegmentum
12	F/54	Yes	Yes	Yes	Yes	Yes	Yes	Borderline	No
13	F/10	Yes	Yes	Yes	Yes	Yes	No	Negative	No
14	F/57	Yes	Yes	Yes	Yes	Yes	No	Negative	No
15	F/73	Yes	Yes	Yes	Yes	Yes	No	Negative	Nonspecific white matter abnormality
16	F/33	Yes	Yes	Yes	Yes	Yes	No	Negative	No
17	F/57	Yes	Yes	Yes	Yes	Yes	No	Negative	Nonspecific white matter abnormality
18	F/27	Yes	Yes	Yes	Yes	Yes	No	Negative	No
19	F/55	Yes	Yes	Yes	Yes	No	No	Negative	Nonspecific white matter abnormality
20	F/46	Yes	Yes	Yes	No	No	No	Negative	Nonspecific white matter abnormality
21	F/53	Yes	Yes	Yes	No	No	No	Negative	No
22	F/40	Yes	Yes	Yes	No	No	No	Negative	No
23	F/28	Yes	Yes	Yes	No	No	No	Negative	Asymptomatic focal right frontal white matter lesion
24	F/34	Yes	Yes	Yes	No	Yes	No	Negative	No
25	M/20	Yes	Yes	Yes	No	Yes	No	Negative	No
26	F/57	Yes	Yes	Yes	No	No	No	Negative	Nonspecific white matter abnormality

### MOG-IgG positivity: CRION vs. RION vs. monophasic iON

**Fig. 3:**
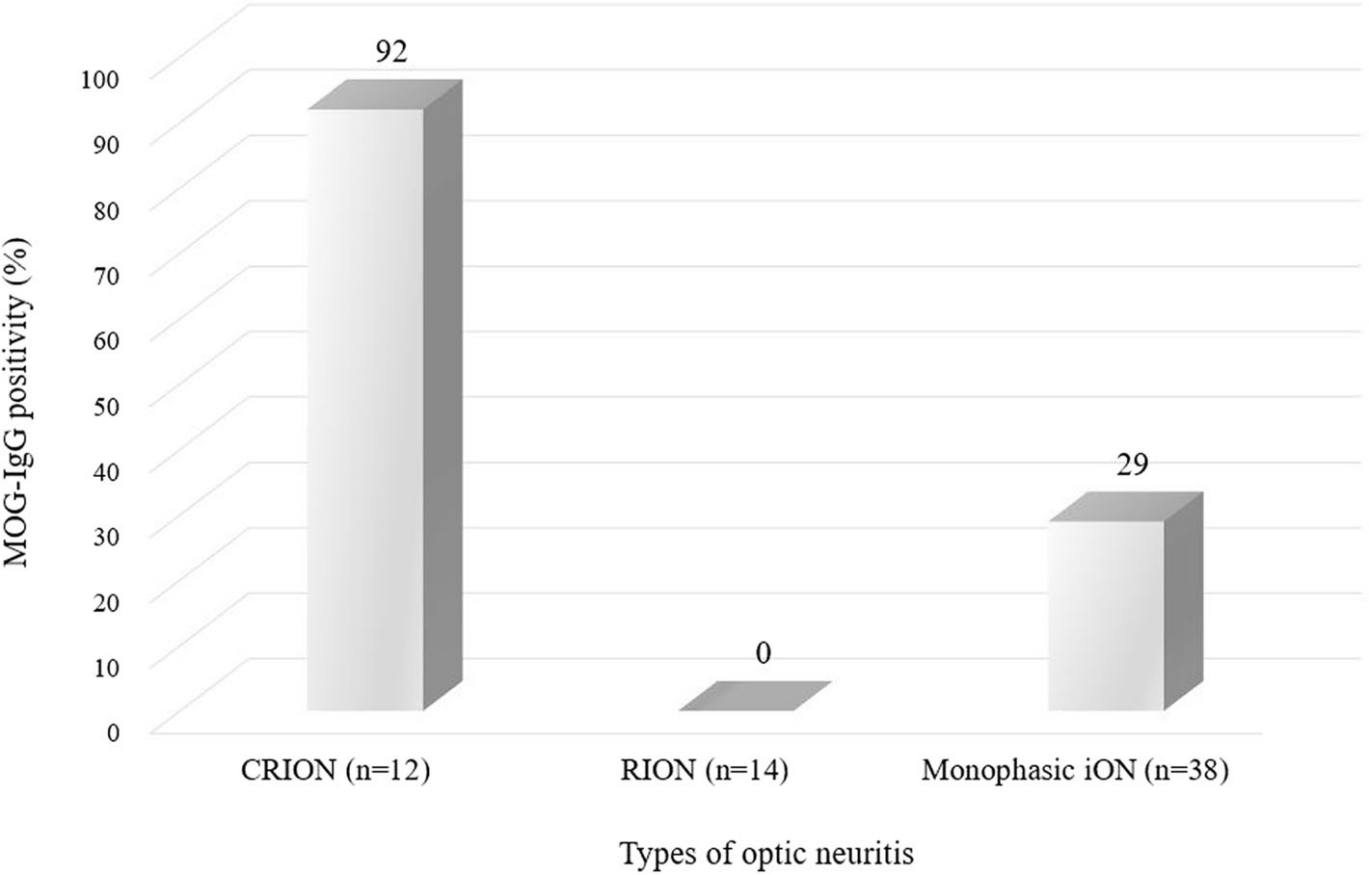
MOG-IgG positivity in chronic relapsing inflammatory optic neuropathy (CRION, *n* = 12), recurrent idiopathic optic neuritis (RION, *n* = 14), and monophasic idiopathic optic neuritis (monophasic iON, *n* = 38). MOG-IgG positivity was 92% in patients with CRION, 0% in patients with RION, and 29% in patients with monophasic iON

Patients with iON (*n* = 64) were classified into three groups according to their courses and characteristics of ON [2, 14, 15]: (1) CRION (*n* = 12), (2) recurrent idiopathic ON (RION) (*n* = 14), and (3) monophasic idiopathic ON (monophasic iON) (*n* = 38). MOG-IgG positivity was 92% in patient with CRION, 0% in patients with RION, and 29% in patients with monophasic iON (Fig. 3).

### Patients positive for MOG-IgG: CRION vs. non-CRION

Of the 11 CRION patients with MOG-IgG (CRION-MOG+ group), seven (64%) were females. The mean age of the first ON attack was 41.4 ± 12.8 years (range 20.8–62.1). Optic disc swelling was present in 33% of the patients. Pain with eye movement was presented in 82% of the patients, and a VA less than 20/200 at nadir was present in 80% of the patients. Perineural enhancement of the optic nerve was present in 71% of the patients.

Of the 52 patients who did not meet the criteria for CRION, 11 tested positive for MOG-IgG (non-CRION-MOG+ group). In contrast to the CRION-MOG+ group who all had relapsing disease courses, our non-CRION-MOG+ patients had only monophasic disease courses. The CRION-MOG+ group experienced steroid-dependent worsening/relapse in the early stages of the disease (2.3 months from disease onset). The CRION-MOG+ group had more frequent ON attacks, and thinner retinal nerve fiber layer and ganglion cell-inner plexiform layer thicknesses, than the non-CRION-MOG+ group. The duration of oral immunosuppressive treatment after first attack did not differ significantly between the groups (Table 2).

**Table 2:** Comparison of clinical features in patients with MOG-IgG-positive isolated optic neuritis according to the disease course

Variables	CRION with MOG-IgG (*n* = 11)	Non-CRION with MOG-IgG (*n* = 11)	*P* value
Age at 1st onset (years)	41.4 ± 12.8 (20.8–62.1)	45 ± 21 (15–70.7)	n.s.
Sex (M:F)	4:7	6:5	n.s.
Presence of the serum IgG1 MOG-IgG (*n*)	11 (100%)	11 (100%)	n.s.
Relapsing disease course (*n*)	11 (100%)	0	< 0.001
Duration of oral immunosuppressive treatment after 1st attack (months)	1.0 ± 1.0 (0–2.8)	2.4 ± 2.8 (0–9.1)	n.s.
Unilateral:Bilateral involvement (*n*)	6:5	6:5	n.s.
Pain with eye movement (*n*)	9 (82%)	8 (73%)	n.s.
Optic disc swelling (*n*)	3/9 (33%)	7 (64%)	n.s.
Visual acuity at nadir (logMAR)	1.5 ± 0.9 (0–2.6)	1.3 ± 1 (0–2.3)	n.s.
Visual acuity less than 20/200 at nadir (*n*)	8/10 (80%)	5/10 (50%)	n.s.
Enhancement of optic nerve on MRI (*n*)	11 (100%)	10/10 (100%)	n.s.
Perineural enhancement of optic nerve on MRI (*n*)	5/7 (71%)	6/10 (60%)	n.s.
Anatomical segment of optic nerve enhancement			
Proportion in orbital area (%)	70.0 ± 26 (30–100)	69.5 ± 30 (20–100)	n.s.
Orbital (*n*)	9/9 (100%)	10/10 (100%)	n.s.
Canalicular (*n*)	3/9 (33%)	4/10 (40%)	n.s.
Intracranial (*n*)	2/9 (22%)	0/10 (0%)	n.s.
Chiasmal (*n*)	1/9 (11%)	0/10 (0%)	n.s.
Pattern of visual field defect at first attack (n)			
Central	5 (45%)	5 (46%)	n.s.
Altitudinal	1 (10%)	3 (27%)	n.s.
Diffuse	5 (45%)	3 (27%)	n.s.
Total follow-up duration (months)	45.7 ± 25.8 (5.8–104.5)	42.6 ± 31.8 (9.1–95.8)	n.s.
Total number of attacks (*n*)	4.1 ± 1.8 (2–7)	1 ± 0 (1–1)	< 0.001
Time interval between 1st and 2nd attack (months)	2.3 ± 2.2 (0.4–7)	–	–
Frequency (total number of attacks/years)	1.7 ± 1.6 (0.3–6.2)	0.6 ± 0.6 (0.1–2.1)	0.023
Visual acuity at final follow-up (logMAR)	0.2 ± 0.7 (−0.1–2.3)	0 ± 0.1 (−0.2–0.2)	n.s.
Visual acuity more than 20/40 at final follow-up	10/11 (91%)	9/9 (100%)	n.s.
Retinal nerve fiber layer thickness (μm)			
Average	65.7 ± 11.5 (50–81)	83.3 ± 12.5 (69–92)	0.040
Superior	80.7 ± 21.1 (52–107)	107.7 ± 24.9 (79–124)	n.s.
Temporal	43.7 ± 8.7 (33–55)	60.3 ± 1.5 (59–62)	0.001
Inferior	81.5 ± 21.8 (51–114)	99 ± 24.8 (71–118)	n.s.
Nasal	60.3 ± 9.0 (46–77)	70 ± 7.8 (65–79)	n.s.
Ganglion cell-inner plexiform layer thickness (μm)			
Average	64.4 ± 6.5 (52–72)	76 ± 5.6 (71–82)	0.002
Minimum	59.3 ± 7.0 (45–70)	68.7 ± 9 (60–78)	0.006
Central macular layer thickness (μm)	242.5 ± 21.2 (215–282)	237.3 ± 22.9 (207–248)	n.s.

*Abbreviations*: *MOG-IgG* myelin oligodendrocyte glycoprotein immunoglobulin G, *CRION* chronic relapsing inflammatory optic neuropathy, *ON* optic neuritis

## Discussion

Previous studies on the clinical manifestations [1], laboratory findings [15], and diagnostic criteria of CRION [2] have suggested that it is a distinct disease entity, different from other IDDs of the CNS. The reported characteristics of CRION (optic neuritis, dependency on steroids, and the absence of AQP4-Ab) are similar to those described in patients with MOG-IgG disease [3, 9]. Nevertheless, the association between these two disease entities has not been fully evaluated, mostly due to the rarity of CRION [2] and methodological issues associated with MOG-IgG assays [11].

In this study, we demonstrated that (1) the vast majority (92%) of our CRION patients (diagnosed according to the current criteria [2]) were MOG-IgG-positive with relapsing courses, (2) relapsing ON patients without steroid dependency (hence not meeting the criteria for CRION) were not positive for MOG-IgG, and (3) patients with MOG-IgG-positive ON who did not have steroid-dependent relapse in the early stage of the disease (about 2.3 months from onset in the current study), had monophasic courses with favorable outcomes after 43 months follow-up. Unlike previous studies on heterogeneous groups of patients with IDDs of the CNS, this study focused on patients with iON and reported on the MOG-IgG status and its association in patients with CRION. The MOG-IgG assay results of our one CRION patient (MFI ratio of 1.56) was considered to be borderline, as her test result was just below the cutoff value of the MOG-IgG assay (1.64). The borderline result is reflective of our strict cutoff value of + 6 SD. Moreover, her test result was also higher than the highest value of the controls (1.23). This patient may be MOG-IgG-positive in a low titer.

Recent studies have reported that patients with MOG-IgG were frequently associated with steroid-dependent recurrent ON and suggested that a subset of patients diagnosed as CRION may be MOG-IgG-positive [2, 5, 16]. Based on a large cohort of patients (*n* = 615) and accurate IgG1 MOG-IgG assay methods, our study showed that most patients with CRION, as diagnosed according to the current diagnostic criteria [2], are MOG-IgG-positive ON with relapsing courses. Our findings support the proposal to consider CRION as a distinct disease entity [4, 16, 17]. In our study, steroid dependency was a key finding that distinguished CRION from MOG-IgG-negative relapsing ON. Moreover, our results suggest that the absence of steroid dependency in the early stages of the disease could be a predictor for long-term non-relapsing disease courses among patients with MOG-IgG. Testing for MOG-IgG may be needed in patients with steroid dependency in the early stages of the disease.

In contrast to patients with NMOSD-AQP4-IgG, most of whom have a relapsing disease course [18], a considerable number of patients with MOG-IgG1-Abs have monophasic disease courses (20–56%, depending on the follow-up duration) [19, 20]. A recent study including 252 MOG-IgG-positive patients reported that immunosuppression for longer than 3 months following the onset attack was associated with a lower risk of a second relapse [20]. Future studies to investigate the effect of early immunosuppressive treatment on the long-term disease courses are needed.

Although CRION patients generally respond well to steroids [4], one of our patients with CRION had a poor visual outcome, presenting VA less than 20/40 at final follow-up. Moreover, in contrast to recent studies reporting that retinal nerve fiber layer thickness is relatively well preserved in patients with MOG-IgG [21, 22], our study showed significant decreases in retinal nerve fiber and ganglion cell-inner plexiform layer thicknesses in CRION patients with MOG-IgG compared to non-CRION patients with MOG-IgG. Cumulative damage led to poor visual outcomes and structural changes in retinal nerve fiber layer permanently. Similarly, other studies have shown that 3 of 8 MOG-Ab-positive patients (38%) had retinal nerve fiber layer thinning [17]; and 12 of 75 patients (16%) had permanent visual acuity of 6/36 or worse at the last follow-up [20]. These findings suggest that patients with CRION, or at least those with high relapse rates, may benefit from long-term immunosuppressant therapy, for the prevention of further relapses and subsequent optic nerve damages. Nevertheless, the need for an immune suppressive treatment in individual patients with MOG-IgG should be carefully decided, as their prognosis of disease can vary widely and even some of them had monophasic disease courses [19, 20].

MOG-IgG is considered to be a pathogenic and disease-specific autoantibody for MOG-IgG-associated disorders [23, 24]. Cell-based assays for MOG-IgG were rarely positive in multiple sclerosis or NMOSD-AQP4-IgG [11]. The clinical, radiological, and pathological findings of MOG-IgG-associated disorders were distinct from those with multiple sclerosis or NMOSD-AQP-IgG [4, 20, 25]. The MOG-IgG titers were related with disease course, which may be helpful for predicting the prognosis of disease [24]. In a transgenic mouse model with endogenous MOG-recognizing T cells, constitutive production of autoantibody against MOG caused experimental autoimmune encephalomyelitis [26]. These findings, together with the results of our study, suggested that the B cell targeted treatments could prevent the further relapses in CRION. However, future studies with larger sample populations are needed to investigate the exact benefit/s of B cell-targeted treatment such as intravenous immunoglobulin G and/or rituximab in CRION.

## Conclusion

CRION, according to the current diagnostic criteria, is mostly a manifestation of the relapsing ON associated with MOG-IgG. Patients with relapsing iON who show steroid dependency in the absence of the AQP4-Ab may need to be tested for MOG-IgG. Among patients with MOG-IgG-associated ON, the absence of steroid dependency in the early stages of the disease may be a predictor for a favorable outcome and a long-term non-relapsing disease course.

## Additional files

Additional file 1: Histogram of the flow cytometry for healthy control (A), MOG-IgG-positive sera with a dilution of 1:200 (B), 1:100 (C), and 1:200 (D). (TIF 160 kb)

Additional file 2: Histogram of the flow cytometry and geometric mean fluorescence (G-mean) ratio for healthy control and patients with chronic relapsing inflammatory optic neuropathy (CRION). (A) Healthy control sera, (B-H) CRION patient sera with positive for MOG-IgG, (I) CRION patient sera with borderline. (TIF 380 kb)

## Abbreviations

AQP4-Abs: Aquaporin-4 antibodies
CNS: Central nervous system
CRION: Chronic relapsing inflammatory optic neuropathy
FACS: Fluorescence-activated cell sorting
iON: Isolated optic neuritis
MOG-Abs: Myelin oligodendrocyte glycoprotein antibodies
MOG-IgG: Myelin oligodendrocyte glycoprotein immunoglobulin G
MRI: Magnetic resonance imaging
NMO: Neuromyelitis optica
NMOSD: Neuromyelitis optica spectrum disorder
ON: Optic neuritis
VA: Visual acuity
