# Supplementary figures and images for: Chronic relapsing inflammatory optic neuropathy (CRION): a manifestation of myelin oligodendrocyte glycoprotein antibodies
Source: J Neuroinflammation. 2018 Oct 31;15:302. doi: 10.1186/s12974-018-1335-x (PMC6208174; doi:10.1186/s12974-018-1335-x)

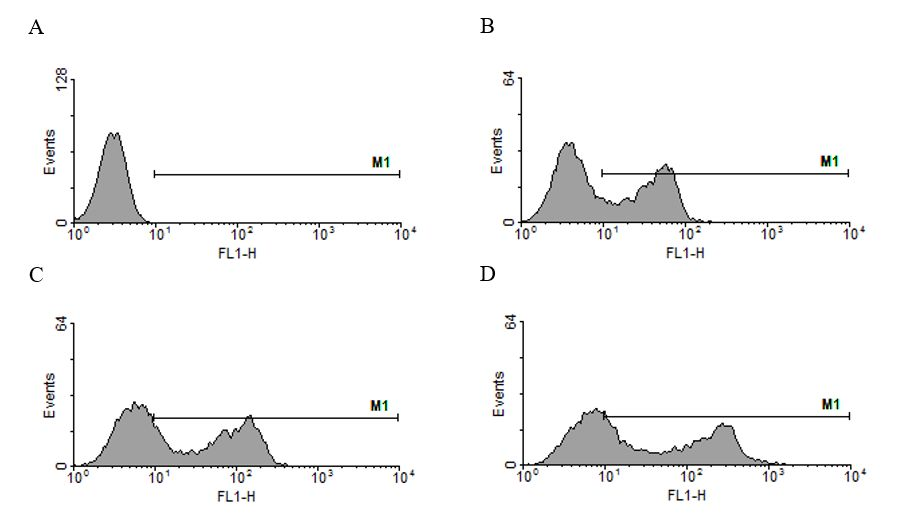

Supplement: Supplementary file 1 — Histogram of the flow cytometry for healthy control (A), MOG-IgG-positive sera with a dilution of 1:200 (B), 1:100 (C), and 1:200 (D). (TIF 160 kb) [file 12974_2018_1335_MOESM1_ESM.tif]

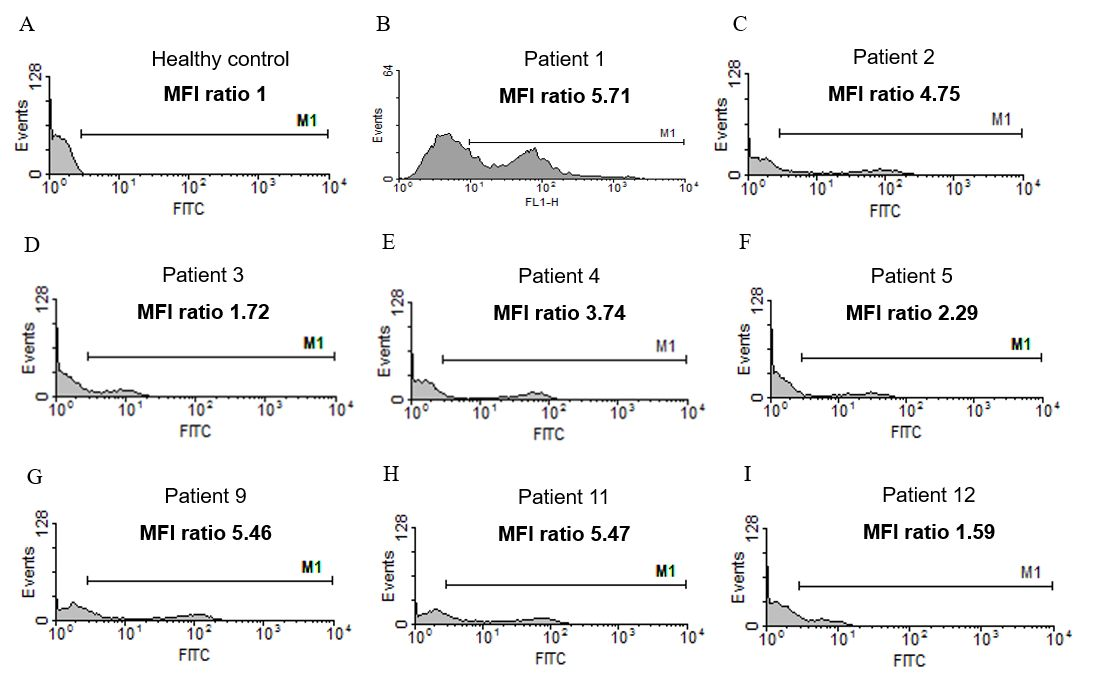

Supplement: Supplementary file 2 — Histogram of the flow cytometry and geometric mean fluorescence (G-mean) ratio for healthy control and patients with chronic relapsing inflammatory optic neuropathy (CRION). (A) Healthy control sera, (B-H) CRION patient sera with positive for MOG-IgG, (I) CRION patient sera with borderline. (TIF 380 kb) [file 12974_2018_1335_MOESM2_ESM.tif]
